# Evaluating An Automated Compounding Workflow Software for Safety and Efficiency: Implementation Study

**DOI:** 10.2196/29180

**Published:** 2021-11-02

**Authors:** Ülle Helena Meren, James Waterson

**Affiliations:** 1 East Tallinn Central Hospital Tallinn Estonia; 2 Medical Affairs Medication Management Solutions Becton Dickinson Ltd Dubai United Arab Emirates

**Keywords:** compounding, medication safety, positive patient identification, gravimetric, automation, closed loop

## Abstract

**Background:**

The forms of automation available to the oncology pharmacy range from compounding robotic solutions through to combination workflow software, which can scale-up to cover the entire workflow from prescribing to administration. A solution that offers entire workflow management for oncology is desirable because (in terms of cytotoxic delivery of a regimen to a patient) the chain that starts with prescription and the assay of the patient’s laboratory results and ends with administration has multiple potential safety gaps and choke points.

**Objective:**

The aim of this study was to show how incremental change to a core compounding workflow software solution has helped an organization meet goals of improved patient safety; increasing the number of oncology treatments; improving documentation; and improving communication between oncologists, pharmacists, and nurses. We also aimed to illustrate how using this technology flow beyond the pharmacy has extended medication safety to the patient’s bedside through the deployment of a connected solution for confirming and documenting right patient–right medication transactions.

**Methods:**

A compounding workflow software solution was introduced for both preparation and documentation, with pharmacist verification of the order, gravimetric checks, and step-by-step on-screen instructions displayed in the work area for the technician. The software supported the technician during compounding by proposing the required drug vial size, diluents, and consumables. Out-of-tolerance concentrations were auto-alerted via an integrated gravimetric scale. A patient-medication label was created. Integration was undertaken between a prescribing module and the compounding module to reduce the risk of transcription errors. The deployment of wireless-connected handheld barcode scanners was then made to allow nurses to use the patient-medication label on each compounded product and to scan patient identification bands to ensure right patient–right prescription.

**Results:**

Despite an increase in compounding, with a growth of 12% per annum and no increase in pharmacy headcount, we doubled our output to 14,000 medications per annum through the application of the compounding solution. The use of a handheld barcode scanning device for nurses reduced the time for medication administration from ≈6 minutes per item to 41 seconds, with a mean average saving of 5 minutes and 19 seconds per item. When calculated against our throughput of 14,000 items per annum (current production rate via pharmacy), this gives a saving of 3 hours and 24 minutes of nursing time per day, equivalent to 0.425 full-time nurses per annum.

**Conclusions:**

The addition of prescribing, compounding, and administration software solutions to our oncology medication chain has increased detection and decreased the risk of error at each stage of the process. The double-checks that the system has built in by virtue of its own systems and through the flow of control of drugs and dosages from physician to pharmacist to nurse allow it to integrate fully with our human systems of risk management.

## Introduction

### Background

A systematic review of the literature from 2020 related to automated compounding technology and workflow solutions for the preparation of chemotherapy concluded that “implementation of chemotherapy compounding automation solutions may reduce compounding errors and reduce costs; however, this is highly variable depending on the form of automation” [1].

**Figure 1:**
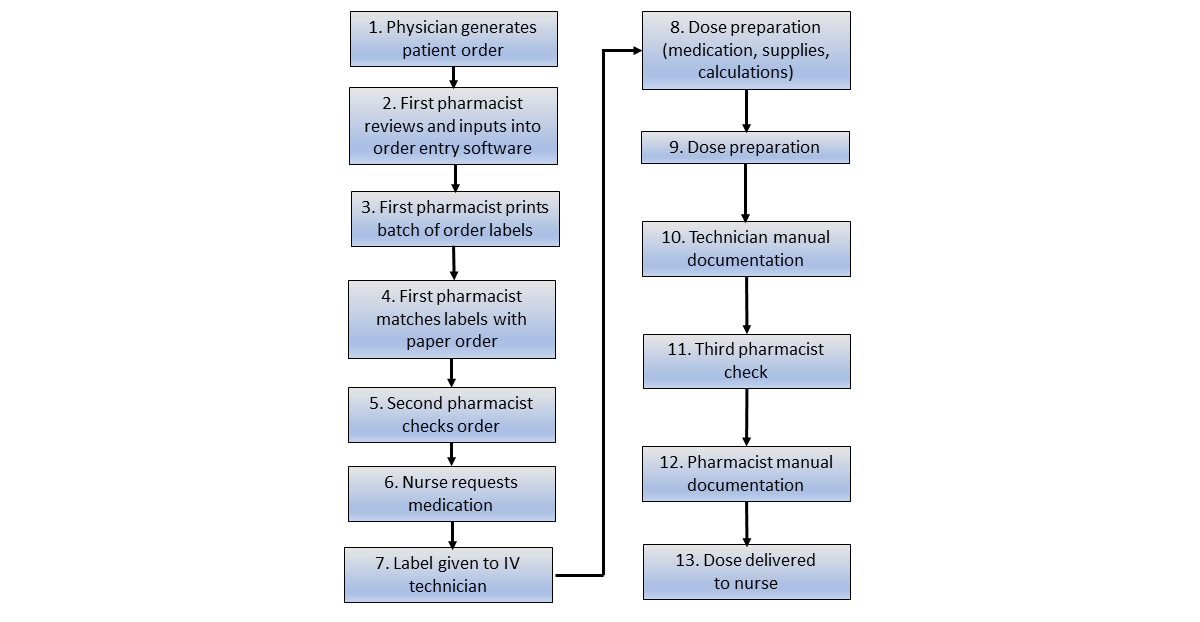
A semimanual intravenous oncology medication chain with safety gaps and productivity choke points. Adapted from Reece et al [3].

In terms of scaling up compounding, managing the entire workflow for oncology therapy management, from prescription via compounding and through to administration, is the most logical solution to the increasing demands that have been seen in both oncology inpatients and outpatients and which will continue to increase in the near future. In fact, one estimate suggested that by 2040, globally, the number of patients requiring at least first-line chemotherapy each year would have increased from a 2018 baseline by 53% (ie, from 9.8 million to 15 million individuals) [2]. An entire workflow management for cytotoxic prescription, production, and administration is desirable because in terms of cytotoxic delivery of a regimen to a patient the chain that starts with prescription and the assay of the patient’s laboratory results and ends with administration has multiple potential safety gaps and choke points (Figure 1).

The possible errors and safety flaws of the aforesaid compounding process in the pharmacy begin with possible errors of transcription at Step 2. Order entry software may have dose-limiting features, which may reduce the risk of dose transcription error, but which may miss area under the curve (AUC) dose reductions in the original order. Without integration into prescription software the risk of “simple” lookalike–soundalike transcription errors also exists. The pressure for delivering multiple patient doses and the fact that the pharmacist is often only present at key stages for technician checks gives Step 3, the pre-emptive printing of a batch of order labels, the potential for causing mix-ups of patient-product labels with incorrect product labeling at Steps 7-10. Besides, under this system the third pharmacist check at Step 11 is of marginal value in a batching process as used ampoules, carriage fluids, and labeled final patient products may be verified but with no guarantee of accuracy nor correct patient-product matching in any unwitnessed steps (highly likely to be Steps 8-10) The issuing of a label checked against the prescription at Step 5 actually *precedes* the physical creation of products that then have these labels applied. This does not follow a logical failure mode effect analysis (FMEA) process [4], where steps involving risk must take place before any final verification checks and the issuing of a label with a unique preparation and patient identification (ID) number. The second pharmacist check at Step 5 similarly precedes the actual creation of patient products and is therefore, in terms of FMEA, redundant.

In terms of the above system’s capacity for maintaining consistent patient-product supply or responding to increased demand there are also several choke points.

Transcription from an unintegrated computerized provider order entry (CPOE) system into the pharmacy compounding system (Step 2) requires the work of 1 pharmacist and replaces clerical work for more useful or appropriate tasks. Steps 3-5 are similarly manual and are perhaps retained as they give a sense of security that the process is under the control of well-qualified pharmacists. Step 11 requires the physical presence of the pharmacist in the compounding clean room, an area that is geographically separate from the main pharmacy suite in our unit. With distractions from other ongoing tasks, both supervisory and specialist, it can be difficult to coordinate between technicians and pharmacists to achieve rapid checks, and this is a major choke point, because it not only slows dispensing of completed patient products, but also causes backlogs as other products cannot be compounded until unchecked patient products are released. Step 12 can delay release of patient products to the nursing unit, as in a compounding system that is unintegrated with administration this is the last point at which the compounding unit has visibility over the medication; therefore, manual documentation must take place before patient products can be transported for administration. This is another clerical action that takes the pharmacist away from higher-value tasks.

Variations of doses or dose adjustments close to the time of therapy due to late-phase AUC adjustments based on the patient’s laboratory results can also cause wastage or require recompounding in rigid systems that require longer loading and setting times.

There is of course a requirement for accuracy of compounding beyond those relating to AUC alterations. Compounding errors may be of magnitudes significant enough to cause direct patient harm, and the problem persists despite advances in workflow management and technological assistance in the compounding space. A recent survey of both pharmacists and pharmacy technicians found that 74% of all respondents were aware of at least one compounding error in the past 12 months, including those discovered in the pharmacy and after dispensing, with incorrect dose or concentration being the most cited error (58%) of all of those discovered [5].

A 2016 study of the implementation of an oncology compounding workflow software solution showed the gravimetric component of the solution catching 797 deviations from acceptable tolerances (<4%) before injection into the final administration intravenous (IV) bag (11,874 preparations in total). Catches at this stage of the workflow are significant, with the possibility of reworking the dose, and the study noted that no deviations were detected at the final weight verification step, with the correct amount of drug accurately injected into the final administration IV bag [3].

The 2020 survey [5] also identified other errors beyond final dosing/concentration, with incorrect base solution identified by 51% of respondents and incorrect reconstitution of a drug in terms of volume or diluent stated by 36%. Furthermore, only 52% of respondents reported that “it is *always* easy to identify with certainty which (and how many) drugs, diluents, and volumes were used when verifying the preparation of each Compounded Sterile Procedure.” It is notable in this respect that the 2016 study identified how a “no-software” FMEA system detected only 1 wrong diluent event, whereas the compounding workflow software solution when integrated into a new FMEA system detected 52 such events. The FMEA risk priority score (severity score × probability score × detectability score) for “wrong fluid selected” in the 2016 study dropped from 567 to 108 after the software application was implemented [3], chiefly because of a significant drop in the risk of detection failure.

The difficulty of error detection during high-risk processes such as compounding and administration of IV chemotherapy is an issue where technology can undoubtedly assist. The emerging evidence related to medication administration at the bedside, a part of the medication delivery chain where currently it has been suggested that at least 38% of all medication errors occur [6], is that introducing a final barcode scan–based check of “right patient–right medication” using patient ID labels and barcoded medications that include the patient’s medical record number and the “order string” pertaining to the patient’s particular regimen and prescription may reduce the overall error rate by as much as 3:1 [7,8]. Without the presence of “nonhuman” confirmatory processes in place it has been suggested that the detectability of administration error falls as low as 2%, and that of dispensing/compounding error versus prescription error falls to 34% [6].

Human FMEA systems for compounding emphasize the double-check of each stage of the process with pharmacist oversight of technicians. It is likely, however, that pharmacists, given their workload, the reduced numbers of qualified staff available against a backdrop of increasing demand on health care services, and the closed nature of the sterile compounding unit, can only be present for “key stages” of the compounding process. For example, the key parts of the compounding (diluent, medication vials, closed system transfer devices, final administration IV bag, and recipe) may be shown to the pharmacist as a “guarantee” of correct constituents for compounding, but given that lookalike–soundalike errors remain prevalent in pharmacies (estimated at 25.9% of all errors) [9] and that in hurried checks the “4-eyes” process may only reinforce error rather than avert it [10,11], this is far from optimal. To this end, a compounding process that has other monitoring processes outside of the assumed presence and infallibility of a second human check is desirable. A compounding workflow software solution that allows for electronic verification and documentation of each preparation from end-to-end with ideally image recognition and capture that can document workarounds, such as “supermarket-style” scanning of the same ampoule several times for multiple vial usage, can give this level of real-world evidence. Equally, rejected patient-medication scans at the bedside could assist us in identifying a little more of the iceberg of this error, as currently the other established methods are very much retrospective because they are based on chart review [12] or reliant on self-reporting, with all its attendant issues [13].

Auto-documentation of medication administration directly into the patient’s record is certainly superior to manual completion of the medication record, because such documentation is commonly delayed or inaccurate as clinicians attend to emergent situations or distractions [14]. Once clinicians return to their documentation after a patient care event, such as medication administration, they often transcribe from memory. Having a secondary nonhuman confirmation of patient and medication matching via barcode scanning would be of huge value for audit even in systems that are transitioning between electronic prescribing and paper documentation of administration to be able to compare scan-library data with manual chart entries.

Gravimetric systems are an integral part of some compounding workflow software solutions, and their main function has been to ensure dosing is within tolerance. A large-scale European study that ran over 4 years [15] showed how a gravimetric system detected a 7.89% error rate (nearly 60,000 errors) for compounded doses outside of tolerance in a total of 759,060 doses of antineoplastic drugs. Over 10% deviations were seen in a mean of 2.25% (range 0.49%-5.04%) and over 20% deviations were seen in a mean of 0.71% (range 0.21%-1.27%) of compounded medications.

Estonia faces the same pressures seen in other Organisation for Economic Co-operation and Development (OECD) countries: an increasing number of patients with cancer, an acute lack of medical personnel, and increasing restrictions on budgets.

Before the implementation of a gravimetric compounding workflow software solution in the pharmacy serving the Oncology Department of the East Tallinn Central Hospital, nurses prepared all cytotoxic medications on the oncology day care unit. The process was entirely unautomated and undertaken under a biosafety hood using closed-system transfer devices. The same nurses who prepared the medications also administered them. The amount of time spent preparing medications detracted from time spent on patient care and there was no comprehensive documentation of the medication regimen. The workload was becoming untenable by 2012 due to increasing complexity of treatments and increasing numbers of patients.

The organization therefore set itself 4 initial goals:

- Increase the number of oncology treatments.
- Improve patient safety.
- Improve documentation.
- Improve communication between the oncologist, the pharmacist, and the nurse.

### Objectives

The overall objective was to show how incremental change to a core compounding workflow software solution has helped the organization meet the above goals, has released nursing time, and has acted as a catalyst to extend medication safety beyond the compounding of medications in the central pharmacy and to the patient’s bedside, where a further technology enhancement has also improved efficiency, documentation, and confidence in the closing of the medication chain with right patient–right medication transactions being verified and documented.

## Methods

### Materials

The following materials were utilized for the solution implemented: a compounding workflow software solution (BD Cato); a prescribing workflow software solution (BD Cato Prescribe); a closed-system transfer device (BD PhaSeal); a barcode medication administration (BCMA) software suite (BD Cato ReadyMed); a handheld user interface on the bedside; and a BCMA-enabled bedside handheld device (Zebra TC56 Mobile Computer).

### Study Design

The establishment of compounding, prescribing, and administration software and solutions was incremental. Each component was, however, essentially undertaken under conditions of a ceteris paribus pre–post study design except the increasing volume of chemotherapy required and delivered, as staffing, physical space, transportation methods, and communication channels between the compounding unit and nursing units remained unchanged. We undertook a qualitative review of the changes made with staff and within our team structure supported by a retrospective quantitative review of compounding production capabilities over 8 years, and an ex-ante and post-ante quantitative review of time required for nurses to confirm patient ID and product match prior to administration of compounded products with the introduction of BCMA capabilities over a period of 4 weeks.

The process of selection of hardware and software for the move from nurse-led to pharmacy-centered compounding was undertaken in the light of the studies above. The final selection was a compounding workflow software solution, along with the continuance of a CSTD and an existing Class A isolator unit. The CSTD was known to nursing staff, so its continuance of use was logical, as it would reduce workflow change.

In the first build, a configuration for the compounding workflow software solution to manage both preparation and documentation was initiated. The suite had initial pharmacist verification of the order, gravimetric checks, and on-screen instructions displayed on the monitor inside the work area for the technician. The user interface requires minimal interactions during the compounding process. The technician simply follows step-by-step instructions. For example, the interface will propose a list of items that includes drug, diluents, and the consumables required to prepare the dose. Automatic dosage calculations are undertaken by the software according to the prescribed regimen. The use of preset regimens was extended as much as possible to increase standardization and reduce divergence from workflow. Scanning of individual components ensures a recipe match for all items including the final administration IV bag. The system also carries hard stops for dosing out of tolerance as per the recommendations of the studies above [6,15]. Besides, the system only delivers a patient-medication label after all the steps of compounding are successfully completed, which makes it ideal for building into an FMEA process, as the steps involving risk are all before the final verification checks and the issuing of the label with a unique preparation and patient ID number, which reduces the risk of administering the medication to the wrong patient. This was fundamental to our later project to allow for right patient–right medication checks at the bedside.

The software records all cancelled mixes, tolerance limit breaches, incorrect item scans, and the resolutions of alerts by the user. Each distinct group of events from first alert to resolution is recorded. Date–time stamps are applied to all of these alerts. Data are continuously collected from the compounding logs and stored locally on an MS-SQL database inside the hospital firewall. Documentation in the central pharmacy of compounding statistics for each preparation is aggregated and assists with forecasting, and each preparation is date–time stamped.

All pharmacy staff are aware of this ongoing collection and analysis of near-miss events. This is important if we want to get as close as possible to “normal behavior” with our data. As with all observational and self-reporting studies, the Hawthorne effect remains a very real danger. The advantage with “passive” data collection, such as that gathered by the software, is that users will not alter their behavior as they might during a time-limited study. This philosophy of consent through thorough understanding of the nature of data collection was later extended to the nursing trial of handheld medication-patient barcode scanning devices.

Integration between the CPOE system and the pharmacy compounding software suite modules was the next intervention undertaken with the addition of the prescribing module, chiefly to reduce the risk of transcription errors. A 2015 study [16] described how near-miss transcription error (NMTE) reporting rates varied between an institution’s formal reporting system built on traditional lines of self-reporting of near-miss and identified-incident reporting and an adapted NMTE reporting mechanism utilizing an error queue within the institution’s order imaging software. The NMTE system described in the study was similar to the video capture component of our compounding solution but in fact did not have an integration between prescription and compounding. However, it is a useful guide to the number of NMTEs that might be avoided through application of a system that eliminates the need for transcribing and removes a risk element from the process. In this study the data collection spanned 92 days during which time about 460,000 medication orders were processed. In total, 1563 NMTEs were reported using the transcription error queue imaging software (0.34%), while only 12 errors were reported (detected) via the formal reporting mechanism (0.003%).

**Figure 2:**
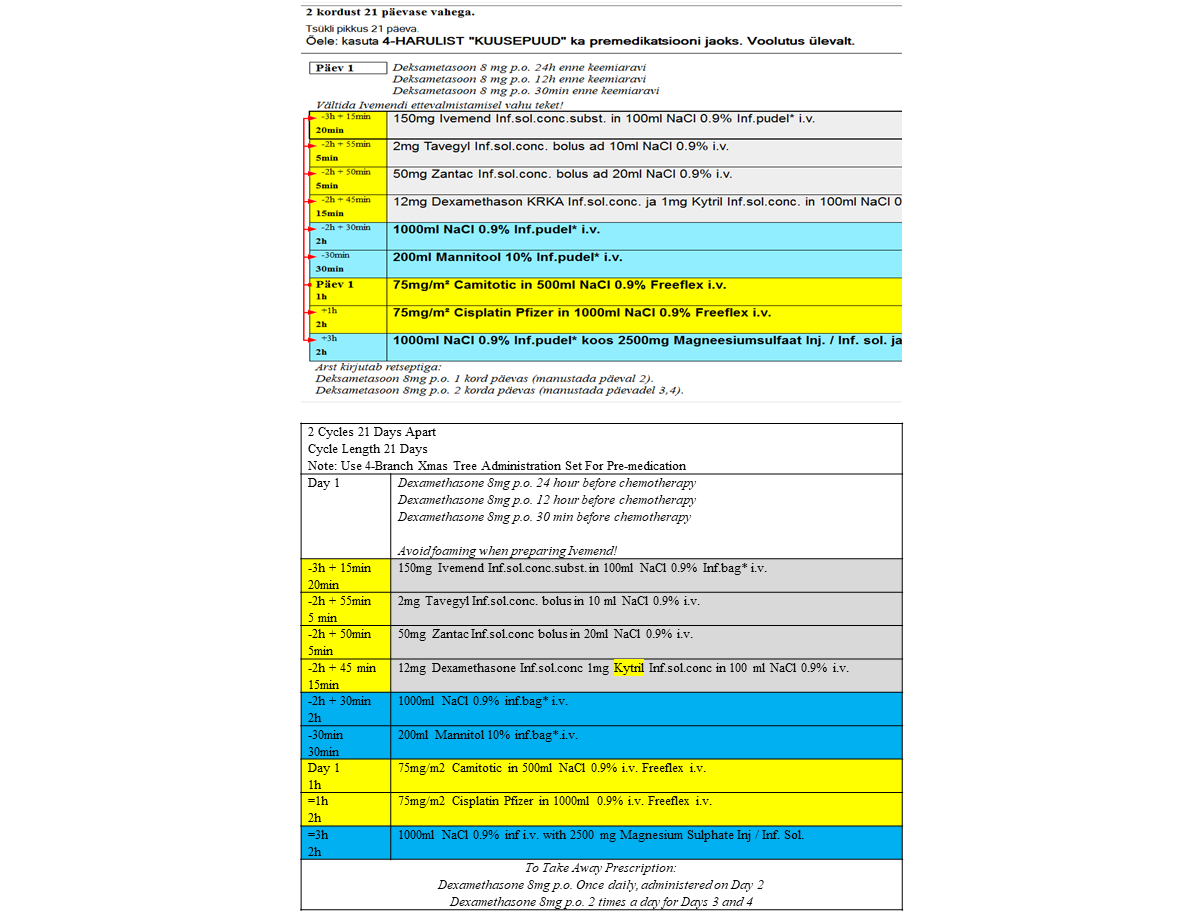
A patient-specific cycle from a regimen as presented to the prescriber, compounder, and nurse via prescription software (with English translation).

The prescribing software also includes hard stops for dosing and automation of calculations for AUC dosing. Physician prescriptions are received electronically into the pharmacy for verification, and if approved, for push communication to the compounding unit. The module also gives access for prescribers to computer-based standardized protocols, which reduce the number of nonstandard regimens requiring creation, can make for a faster regimen build, and automatically calculate doses and creates preparation guidance. A typical regimen is shown in Figure 2.

The compounding library itself was created by, and is updated and confirmed by, the Pharmacy and Therapeutics Committee. A key update that requires regular review is any changes in the specific gravity of core medications as this will impact on the gravimetric check.

**Figure 3:**
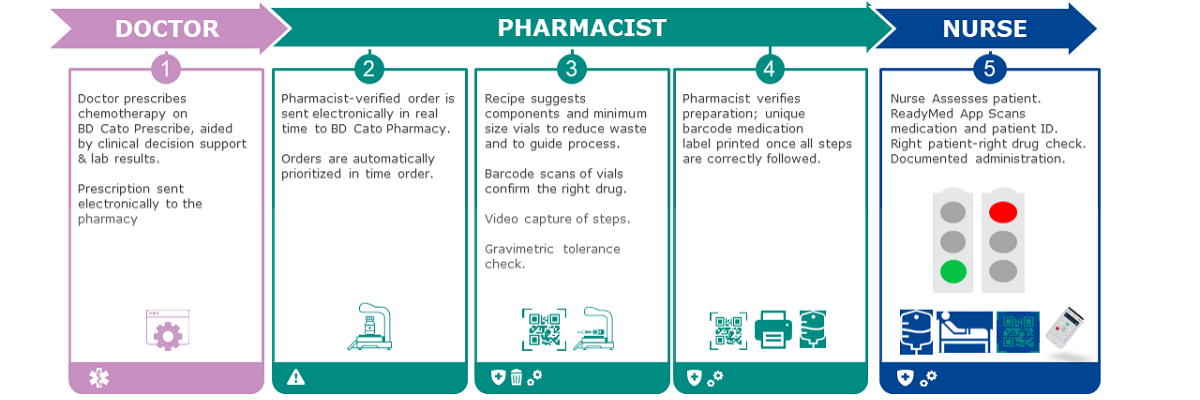
Extension of the patient-medication matching solution from prescribing to the compounding unit and to the bedside via ReadyMed.

The postimplementation workflow (Figures 2 and 3) had changed substantially from the “classic” manual compounding unit workflow described in Figure 1.

We collected data on throughput and set a key performance indicator for reducing time of preparation as a response to receiving no increased full-time employee (FTE) headcount and the need to meet the unit needs, which were forecast to increase at 10%-12% per annum. At the preimplementation stage, the compounding team in the pharmacy, using manual techniques, was averaging ≈6 minutes per preparation. The time was calculated as a mean average of the time taken from the beginning to the end for the compounding of individual 1-ingredient products, with the below steps:

1. Pharmacist reviews and inputs into the order entry software (measurement starts).
2. Pharmacist prints order labels and matches labels with prescription.
3. Second pharmacist checks labels versus order.
4. Technician takes labels and prepares dose: medication selection, supplies and diluents, calculations.
5. Technician documentation.
6. Final pharmacist check of the product.
7. Pharmacist documentation of the product being ready for dispatch to the nursing unit (measurement stopped).

No metrics for mixing by nurses before implementation were available, as their role was split between preparation and administration.

Wastage was addressed by taking advantage of reissuing options in the software, and through the activation of an advisory within the software that proposes the use of a drug vial size that will result in the least amount of waste for the prescribed dose to be compounded. Analysis of each preparation’s data, and aggregation of these data, assisted us in managing and optimizing the inventory, monitoring drug wastage, and measuring productivity.

The second stage of the project involved an extension into the inpatient unit with the deployment of wireless-connected handheld barcode scanners. This allowed us to take advantage of the patient-medication label on each compounded product via the BCMA device (handheld at the bedside).

The move to BCMA was seen as a desirable part of our build for patient safety and efficiency, and the BCMA administration system we envisaged was to feed directly from the prescription module software and to obtain its products for administration from an integrated pharmacy module.

The BCMA handheld interface was initially only available in English but was intuitive enough for the launch; an Estonian language product was available later in the project. The process of scanning the patient for positive patient ID, and then scanning the product to be administered triggers a matching of patient and product information from the compounded product’s barcode to the patient’s ID and to the prescription via the BCMA software and at the interface of the prescribing server. There was and remains a regular process of engagement with nursing leadership and clinical educators to introduce functional changes to the workflow. Acceptance of the new process was good. The new workflow (Figure 3) has replaced a large amount of manual activity by nursing staff and should help to reduce the risk of medication errors by a substantial degree given the literature findings above. The 2 nurse or “4-eyes” check is not a common practice in our facility and is not mandated in Estonia. Prior to the software implementation, the physician printed, verified, and signed the therapy plan, and handed it over to the nurse who matched the paperwork with the product and then with the patient. The process was laborious and charts and order sheets were at risk of being mislaid and were commonly not readily at hand to be aligned and checked against each other.

For audit purposes the processing time of each patient administration can be calculated from date–time stamps on the handheld device, but before implementation data had to be gathered manually. Before and after the implementation of the ReadyMed solution, staffing remained unchanged with an average of 3.5 nurses on unit duty, 3 pharmacy technicians, and 2 pharmacists. The inpatient unit remained at an 8-bed capacity. Walking time between the nursing workstation, where initial checks of the received compounded products is performed, and the patient rooms was unchanged at 10.8 minutes per day of “travel time.” By this point the pharmacy compounding unit was producing ≈14,000 cytotoxic products per annum.

The preimplementation observation was undertaken with consent from nursing staff on a daily basis, as personnel changed on each shift. It was made clear that personal performance would not be identifiable in quantitative results, although data would be continuously collected by the devices. A short timeframe (4 weeks) was deliberately applied for the BCMA ex- and post-ante review to reduce the risk of data distortion arising from increased throughput of patients and the rising number of products compounded and dispensed for administration. This period was long enough to ensure “capture” of all nursing staff during both introduction and training periods. The study type was a pre–post design in that all other factors were ceteris paribus including the staff involved (all nursing staff used the handheld scanner and had used the traditional paper-based method extensively, and all had equal amounts of training and exposure to the new system).

The mean batch of medications to be given by each nurse per shift, mean averaged over the working year both before and after the handheld scanner implementation, was 15 items (SD 2.7). Quantitative measurements of time taken to process medications in each system were therefore based on an average of total time per 15-item groupings rather than time per single-item measurement to reduce the risk of bias from one-off measurements or from possible clusters of “simple” regimens and single items, or of additionally complex regimens.

### Study Procedure

The data were patient anonymized, and no personal information items such as clinician ID, hospital number, gender, name, date of birth, diagnosis, or other identifiable material were recorded for analysis.

BD Clinical Management and Global Customer Service were engaged to optimize the solution and BD Medical Affairs were requested to undertake a deeper analysis of the data. The medical affairs department of BD operates as a distinct arm outside of the commercial operations of the company.

### Inclusion Criteria

All cytotoxic infusions compounded from within the oncology formulary (and therefore identifiable in terms of medication name, dose, and duration as per cycle usage over the period) were included in the study. These included weight-based and non-weight–based infusions and body surface area–based infusions.

### Exclusion Criteria

Infusions that did not require compounding such as flush bags and preregimen, premixed hydration infusions that do not pass through the compounding unit were excluded from the study.

## Results

Our forecasts for growth in both patient throughput and the requirement for compounded oncology medications were reasonably accurate. In fact, growth has been 12% overall, with more than 16,000 patient visits per year (outpatient, daily clinic, and inpatient short stay). Compounding production has met this increase without an increase in FTE headcount (Multimedia Appendix 1).

Despite this increased load and unchanged FTE staffing, there was an overall reduction in compounding time of 35% using the same start and endpoints applied in our measurement of preimplementation checks and compounding times (from “order enters system” through to “product available for delivery to nursing unit”). We believe there are savings in improved management of remnants, but quantifying this would be difficult without a full accounting of pre- and post-implementation ampoule usage per comparable volumes of prescriptions compounded. We do not currently have these data available.

The project using the ReadyMed handheld barcode scanning solution showed substantial nurse time savings within a relatively short period. Within 2 weeks a mean average time of 41 seconds (0.697 minutes) was required for each product–patient matching and verification of the order by the system. When calculated against our throughput of 14,000 items per annum, we saw considerable nurse-time savings, as shown in Tables 1 and 2.

For the qualitative component of the BCMA study we identified the following categorizations of statements with an incidence of above 80% (8/9, 89%) in responses after 2 weeks of use of the handheld scanning devices (Textbox 1). The overall satisfaction was measured by means of a numerical scale. Categorization by statements with responses with an incidence of greater than 80% (8/9, 89%) was undertaken. Given the small sample size, this is as much complexity for analysis as could be achieved. The nursing workforce is very small.

**Table 1:** Nursing time saved per day.

Statistics	Time for each daily batch (minutes per nurse)	Number of medications (each processed daily batch per nurse; N=321)
Mean (SD)	10.476 (10.666)	15.286 (7.191)
Median	7	14

**Table 2:** Sensitivity analysis on Q1 and Q3 and range for 14,000 compounded medications scenarios.

Parameter	Verification speed (minutes per item)	Time saving per item (minutes)	FTE^a^ gain per 14,000 items PA^b^ assuming 225 days of work PA per FTE	Nursing time saved per day (hours and minutes per 14,000 items)
Mean	0.697	5.303	0.425	3 hours and 24 minutes
Min	0.222	5.778	0.46	3 hours and 41 minutes
Max	1.875	4.125	0.33	2 hours and 38 minutes
Q1	0.316	5.684	0.45	3 hours and 38 minutes
Q3	1.000	5.000	0.40	2 hours and 11 minutes

^a^FTE: full-time employee.

^b^PA: per annum.

**Grouping and statement categorization with incidence of greater than 80% (8/9, 89%) in responses (N=9).**

Perception of safety/confidence

- “Easier to identify patient”
- “Chance of medical error was less”
- “Device prompts user to identify the patient”
- “Notification if you try to administer the wrong drug”

Usability

- “Easier to follow the drug chart”
- “Saves time for documentation”

Documentation

- “Local language would increase the use”
- “With one scan it marked who administered, which drug, and at what time”

## Discussion

In classic FMEA planning [4], for any high-risk activity, and particularly for those with a high risk of “low-chance or no-chance” of detection of error, the activity is broken down into a number of steps, each of which can mitigate, correct, or annul any error in the previous steps. The addition of prescribe–compound–administration software solutions to our oncology medication chain has increased detection, and decreased the risk, of error at each stage of the medication chain. What is significant in the process that we describe is that this took a considerable period (over 8 years) to reach its end points.

We could see that the issues of clerical tasks most heavily impacted the compounding unit, and given the limited budget, our 4 goals to increase production and patient safety were the most pressing demands. We focused initially on the workflow described in Figure 1. The benefits for productivity were significant with the choke points at Steps 7, 10, and 12 being removed. In this interim period, Steps 3 and 4 moved to the end of the process where they could be of real value in the FMEA approach, and Steps 8 and 9 were made safer by implementing gravimetric checks and hard stops, as well as guidance from the “recipe” screen. Our outcomes matched those of the study by Reece et al [3] in terms of no deviations being detected in the final weight verification step, a “good catch” rate of less than 4% for out-of-tolerance compounded medications before their injection into the final administration IV bag, and injection of the correct amount of drug accurately into the final administration IV bag in all compounded final products, as verified by the software’s documentation of the process through video capture and recording of gravimetric data.

However, at this stage it was not possible to avoid the transcription risks in Step 2. For a considerable period the final pharmacist’s check therefore had to focus on the paper prescription for validation of the final compounded product to reduce the risk of checking against an invalid transcription, though this was supported by the documentation of the compounding process through video captures and process recording.

As noted previously, it had been hard to coordinate between technicians and pharmacists to undertake key point checks. With the new system, however, technicians did not need to wait for the pharmacist’s physical availability for checks, as these can be made via the process recording. This allowed for faster release of products from the clean room and the release of this choke point on throughput.

We could see from our road map that investment in the prescribing module would achieve improved patient safety given the findings of the NMTE study of 2015 [16] and it would again reduce the clerical load significantly. Step 2 of Reece et al’s [3] semimanual oncology medication compounding chain and the risk of NMTE were essentially eliminated with the addition of the prescribing to compounding modules. The elimination of the CPOE to pharmacy system transcription requirement also ended the requirement for the physical presence of the pharmacist in the compounding clean room, as orders now feed directly from the CPOE to the “recipe” screens.

Manual documentation was also eliminated at this point, but printing of the regimen was still required as the administration roadblock remained. The system, as an interim measure, still enabled us to print patient-medication orders and match attendant medication labeling against patients’ case notes, and for its identification against patient IDs; however, as productivity and demand for compounded products had increased significantly (Multimedia Appendix 1), it was clear to us that administration also needed to move from a paper to electronic form to be in harmony with prescribing and compounding.

We recognize the limitations of the approaches we have undertaken. One key issue was the protracted length of time that the total study took place over, with ongoing changes to regimens and addition and deletion of medications, and the lack of preautomation data that were available to us for comparing and contrasting. Most of the key elements including staffing numbers, the physical environment, and the supply chain have essentially remained unchanged over this period, which has allowed us to accept the assumption that the changes in performance have been related to the introduction of the 3 solutions. This would not necessarily be the case in most units or facilities with fluctuating headcounts, physical unit changes, and changes in supply processes. This said, while the length of time over which the compounding solution was reviewed should reduce the risk of bias, as it is distinctly long-run data (please note the small drop-off in production in 2020 due to the COVID-19 emergency), there is a risk that the benefits of BCMA which we saw are not reflected on such a significant scale in larger units or in specialist patient subpopulations.

There are very few oncology units in Europe or globally that have a similar experience to us in the area of BCMA handheld devices for IV medication administration. It would be a natural progression of this study to a multicenter compounding study, including units with larger headcounts, and in environments with different medication delivery services to the oncology unit.

On a national level the project and its outcomes have been significant. The ability to see a patient’s treatment history with a single click fits well with Estonia’s vision of digital hospitals with entirely paper-free documentation. We are now documenting any side effects through the prescribing software, and we are expanding on features such as doctors and pharmacists being able to leave specific administration cautions or notes for nurses to be picked up at the patient ID and medication ID stage of administration. The next stage of the project should be to extend it across a larger hospital campus system and networking the main hospital with our partner hospitals. This expansion in the region is important as it may drive health technology vendors toward accelerating product localizations. Adapting interfaces to local language increased acceptance of the new technologies in our experience, although even with English interfaces initially being used staff were enthusiastic about the changes, and their individual learning curves were not influenced by age or general technology acceptance, but by their acknowledgement of the advantages the systems gave, particularly in terms of protecting patients. We found that the technology was quickly adopted by all staff after only a few days. The qualitative survey of nurses was encouraging in this respect with statements such as “chance of medical error was less” and “notification if you try to administer the wrong drug” being given as positives.

Studies of error in health care have found that most serious errors occur during the execution of treatment, with “performance-level failures outweighing rule-based or knowledge-based mistakes” [17]. For this reason, we are very positive about the presence of hard stops rather than advisories in the software for prescribing, compounding, and administration for both dosing and medication components and patient ID. Our staff are highly skilled and experienced but are human.

## Appendix

Multimedia Appendix 1 Total oncology medications compounded per annum. Implementation of the compounding workflow software solution took place in late 2012.

## Abbreviations

AUC: area under the curve
BCMA: barcode medication administration
CPOE: computerized provider order entry
FMEA: failure mode effect analysis
FTE: full-time employee
NMTE: near-miss transcription error
