# Supplementary figures and images for: Evaluating An Automated Compounding Workflow Software for Safety and Efficiency: Implementation Study
Source: JMIR Hum Factors. 2021 Nov 2;8(4):e29180. doi: 10.2196/29180 (PMC8596227; doi:10.2196/29180)

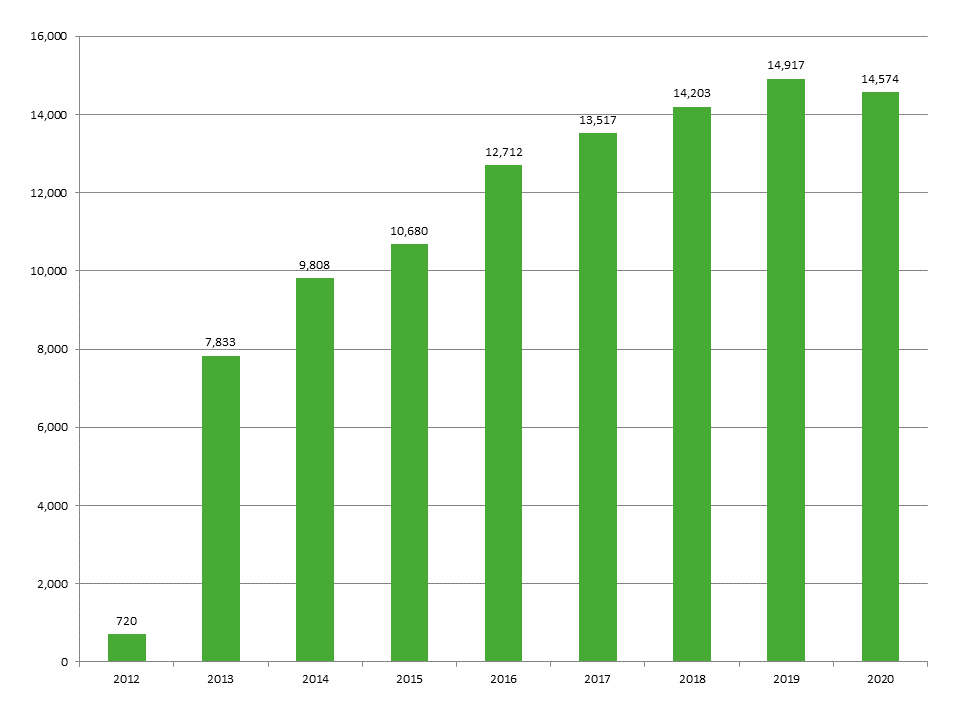

Supplement: Multimedia Appendix 1 [file humanfactors_v8i4e29180_app1.png]
